# Supplementary material for: The causal relationship between the human gut microbiota and pyogenic arthritis: a Mendelian randomization study
Source: Front Cell Infect Microbiol. 2024 Nov 26;14:1452480. doi: 10.3389/fcimb.2024.1452480 (PMC11629706; doi:10.3389/fcimb.2024.1452480)
Supplement: Supplementary file 1 [file DataSheet1.zip › mendelian test/ebi-a-GCST90027765.csv_forest.pdf]

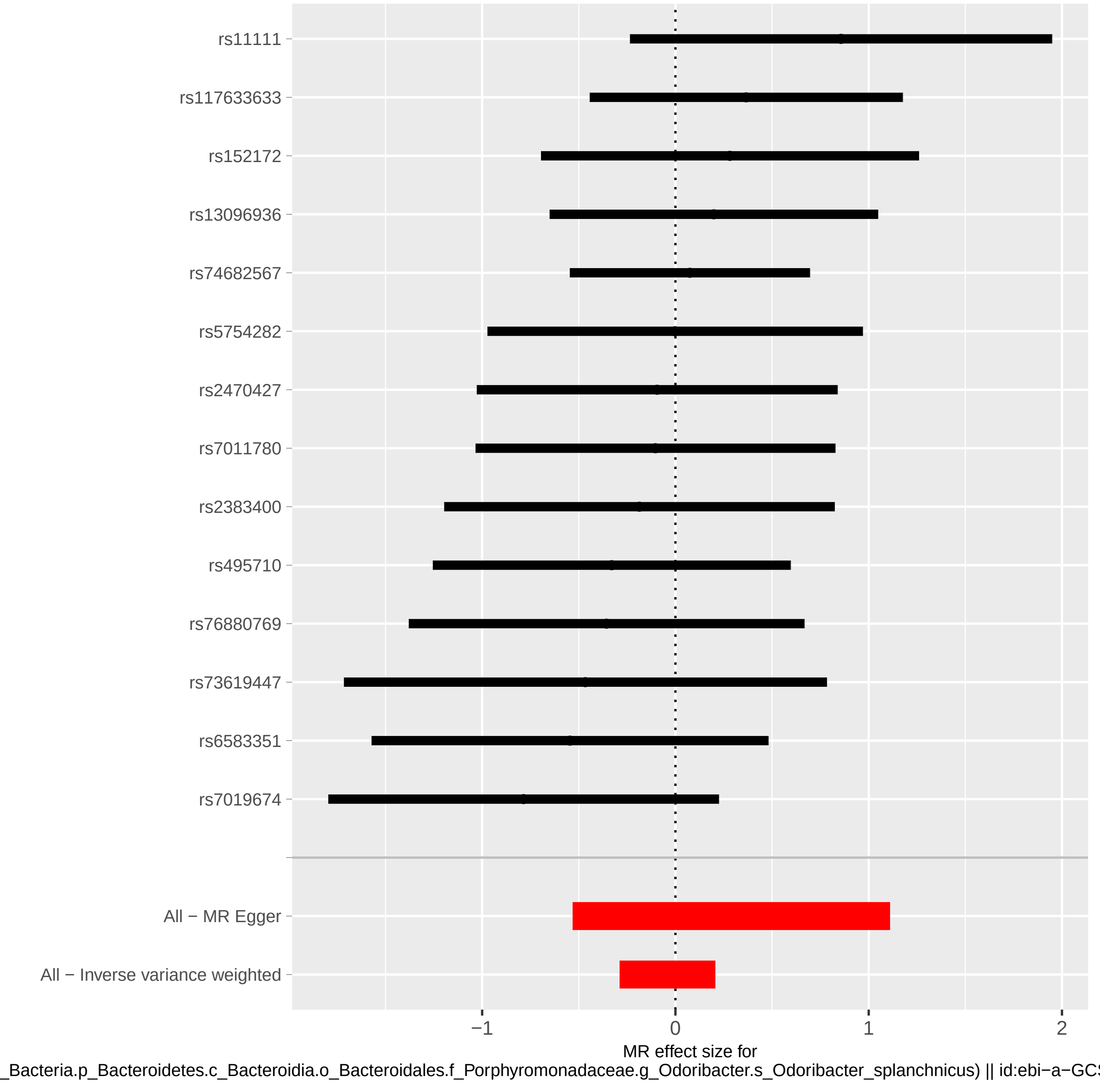
rs11111
rs117633633
rs152172
rs13096936
rs74682567
rs5754282
rs2470427
rs7011780
rs2383400
rs495710
rs76880769
rs73619447
rs6583351
rs7019674
All − MR Egger
All − Inverse variance weighted
−1
0
1
2
MR effect size for
_Bacteria.p_Bacteroidetes.c_Bacteroidia.o_Bacteroidales.f_Porphyromonadaceae.g_Odoribacter.s_Odoribacter_splanchnicus) || id:ebi−a−GC
